# Weighted Bayesian Belief Network: A Computational Intelligence Approach for Predictive Modeling in Clinical Datasets

**DOI:** 10.1155/2022/3813705

**Published:** 2022-07-20

**Authors:** Shweta Kharya, Edeh Michael Onyema, Aasim Zafar, Mohd Anas Wajid, Rockson Kwasi Afriyie, Tripti Swarnkar, Sunita Soni

**Affiliations:** ^1^Bhilai Institute of Technology, Durg 491001, India; ^2^Department of Mathematics and Computer Science, Coal City University, Enugu, Nigeria; ^3^Adjunct Faculty, Saveetha School of Engineering, Saveetha Institute of Medical and Technical Sciences, Chennai, India; ^4^Department of Computer Science, Aligarh Muslim University, Aligarh 202002, India; ^5^Department of Information and Communication Technology, Dr Hilla Limann Technical University, WA, Ghana; ^6^S'O'A Deemed to Be University, Bhubaneswar 751001, India; ^7^Bhilai Institute of Technology, Durg, 491001, India

## Abstract

There are growing concerns about the mortality due to Breast cancer many of which often result from delayed detection and treatment. So an effective computational approach is needed to develop a predictive model which will help patients and physicians to manage the situation timely. This study presented a Weighted Bayesian Belief Network (WBBN) modeling for breast cancer prediction using the UCI breast cancer dataset. New automated ranking method was used to assign proper weights to attribute value pair based on their impact on causing the disease. Association between attributes was generated using weighted association rule mining between two attributes, multiattributes, and with class labels to generate rules. Weighted Bayesian confidence and weighted Bayesian lift measures were used to produce strong rules to build the model. To build WBBN, the Open Markov tool was used for structure and parametric learning using generated strong rules. The model was trained using 70% records and tested on 30% records with a threshold value of minimum support = 36% and confidence = 70% which produced results with an accuracy of 97.18%. Experimental results show that WBBN achieved better results in most cases compared to other predictive models. The study would contribute to the fight against breast cancer and the quality of treatment.

## 1. Introduction

Breast cancer has become one of the major causes of untimely deaths among women [1–3]. There is an exponential increase in the cases of breast cancer globally [4]. According to a 2018 report on breast cancer statistics, there are about 1, 62,468 new registered cases, and more than 87,090 deaths because of breast cancer [5]. As the number of breast cancer patients increases, the need for its detection on time becomes sacrosanct [6]. In order to build and improve breast cancer diagnostic system that will help domain experts to make more effective treatment strategies, data mining techniques plays a vital role. Data mining involves a bunch of advancements for a variety of objectives such as regression, classification, association rules, and clustering under supervised and unsupervised learning [7, 8]. In this study, the focus is on classification techniques applying to breast cancer disease and to build and strengthen the decision-making process. Here, exploration is done on the Bayesian belief network as a classifier in which structure learning is carried out using strong rules generated after applying weighted association rule mining and weighted Bayesian confidence and lift concept.

As attributes have a major role and effect in determining any disease so the weighted concept is introduced to attributes in which higher weights are assigned to the most influenced attributes. To enhance diagnostic results on the basis of accurate prediction and to aid physicians to make significant decisions, there is a demand to develop computer-aided diagnosis systems [9]. An attempt is made in this regard in the current study. This study employed Weighted Bayesian Belief Network modeling approach to find the significance and relationship between different attributes of breast cancer using a dataset extracted from the UCI Machine Learning Repository. The contributions of this study are: discovery of attributes with higher significance by assigning weights using an automated method; Analysis of the association between different attributes to generate rules; Applying Weighted Bayesian confidence and weighted Bayesian lift measures to generate strong rules; and development of WBBN using these strong rules as a computational intelligent predictive model for diagnosis of breast cancer disease. The paper is organized as follows: Section 2 contributes towards related work on the technique used.  Section 3 presents the overall methodology and materials used in building the Weighted Bayesian Belief Network.  Section 4 discusses results obtained from an experiment done with a breast cancer dataset.  Section 5 is a comparative study of the proposed model with various other clinical datasets and also proposed model is compared with different classifiers. While Section 6 concludes the proposed method and illustrates some future courses of action.

## 2. Related Work

### 2.1. Bayesian Belief Network and Its Learning and Construction

Bayesian Belief Network (BBN) is a robust tool for depicting the structure having graphical dependency among variables directly and inherently [10]. To reason in uncertainty, model complexity and nonlinear relationships between attributes, the most promising tool are the Bayesian belief network [11]. In the contemporary study, the application of BBN has become more frequent in a clinical survey by showing its skill to manage useful information related to prediction which is previously unknown related to prediction, detection, and determination of clinical results [10, 11]. The BBN formula can be expressed as joint distribution of conditional probabilities indicated by the following equation fd1:

(1) $$\begin{array}{c} P\left(x_{1},x_{2},\ldots x_{n}\right)=\prod_{i=1}^{n}P\left(x_{i}|Pa\left(x_{i}\right)\right). \end{array}$$

In BBN, firstly the objective of the structure learning is to identify the network's topology or geometrical representation of the network to display the relationships between the nodes of dataset attributes or variables. Secondly, the aim of parameter learning is to find quantitatively how a node is related to its parent nodes i.e., whether two variables or nodes are dependent or independent [12]. The construction of BBN can be done using two methods as Construction using expert knowledge [13] and construction using automatic learning [14]. In this paper [15] the authors designed an automated model using a Bayesian Belief Network, one most viable option for representing the relationships between expert's diagnoses results. Previous studies like [13] used Genie software to build a Bayesian network based on manual construction and automatic learning with a significance in a wide range of areas in health services research like clinical research, and medical decision making. The study by [16] worked on kidney transplantation and the prediction of its graft survival ratio. The study revealed that the selection of proper predictors can strengthen any predictive model with a potential solution using machine learning approaches. Also, a novel method using the Bayesian networks was used to classify two types of malignancies with a training dataset of 366 records and having an accuracy of 93% [17]. While a study by [18] proposed a framework known as Fuzzy based Bayesian network for heart disease which showed an accuracy of 84%.

### 2.2. Weighted Association Rule Mining

Here, stress is provided on weighted association rule mining as conventional mining of rules is based only on the framework of support and confidence for the findings of frequent item sets with the assumption that all items are equally dominant. But in the medical field, researchers have different ways of thinking and requirement. The importance of rules cannot be only based on the database but it depends on quantitative aspects and qualitative aspects also. So weight can play a significant role to represent knowledge for the medical attributes in the dataset. Recent work by [19] proposed a weighted associative classifier (WAC) of which an automated weight assignment method known as Maximum Likelihood Estimation theory was used to compute weights of all attribute of various UCI datasets like heart, hepatitis, cancer, Pima Indian, liver disorders, and then new framework using weighted support and weighted confidence. Also, [20] used a keyword-based weighting scheme to extract the more impacted features from the database to build the model to find the accurate disease. A disease comorbidity prediction was highlighted in [21]. It also includes an exploration of the associations between disease and comorbidity patterns based on Electronic Health Record clinical data and biological data.

## 3. Methods and Materials

**Figure 1:**
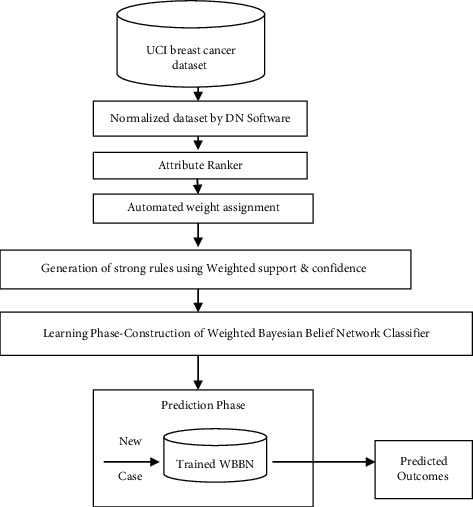
Workflow diagram of the Proposed Model.

The methodology of the proposed research work is shown step by step using workflow diagram as in Figure 1. It shows the processes that were followed accessing the dataset and also training the model and testing its ability to make the required predictions. More details are shown in Section 3.1 and Section 3.2.

### 3.1. Dataset

The study made use of extracted breast cancer dataset from the University of California Irvine machine learning repository accessed via LUCS-KDD DN software. The breast cancer dataset contains 699 clinical records. Each record is populated with nine attributes and one class label. Out of these 699 patient's records, sixteen instances contain values which are missing, so the instances containing missing values are automatically thrown away from the dataset [22]. The actual dataset on which work is done consists of 683 records. The tenth attribute column contains the binary responses of each clinical record. In this dataset, 66% of samples are benign cases, and 34% are malignant samples. The dataset to work upon is taken from the normalized breast cancer dataset in.num format on the UCI machine learning repository website [23]. Following is the set of attributes with discretized values as shown in Table 1.

### 3.2. Proposed Methodology

The Weighted Bayesian Association Rule algorithm showing methodology was already designed in the paper [24]. The whole research carries out on the basis of this proposed algorithm to build an intelligent system and to develop any intelligent system proper steps should be figured out [25]. Using that algorithm important Weighted Bayesian Association rules are mined to build the model of the Bayesian network. According to the algorithm first of all an automated rank based weight assignment technique is applied to assign weights to attribute, and value pair. Then, extraction of rules between two attributes association rules, multi attributes association rules, and class association rules using weighted support and weighted confidence is done. Based on this, rules are generated. Now based on the weighted Bayesian confidence and weighted Bayesian lift strong rules are generated and using these rules Bayesian model is built.

### 3.3. Weighted Approach Using Bayes theorem Based on Ranking Method

The current framework consists of three phases for weight assignments to attributes. In the first step, the attributes are ranked on the basis of the occurrence of the attribute, and value pair in the above dataset. And, then weights are assigned based on probability theory. The attributes are further transformed into an attribute set of the {attribute, value} pair, attribute rank and attribute weight as shown in Table 2.

To generate Table 2 following the procedure is carried out.

Counting is done on the attribute, value pair with “yes” and “no” class labels in the whole dataset.

On the basis of counting, rank is assigned i.e., greater the counting, higher rank is allotted for all attribute, value pair with both class label values.

Here, weights are calculated only for attributes with class label = “yes” because the main focus is on the attribute, value pair with malignant cases (yes) using the Bayes theorem.

#### 3.3.1. Bayes theorem

In statistics, Bayes's theorem describes the probability of an event, based on prior knowledge of conditions that might be related to the event. Bayes's theorem is stated mathematically as the following equation fd2:

(2) $$\begin{array}{c} P\left(\frac{H}{E}\right)=\frac{P\left(E/H\right)*P\left(H\right)}{P\left(E\right)}, \end{array}$$

where H and *E* are events.

### 3.4. Weighted Association Rule Mining

Automated weight assignment using Bayes theorem was applied in the breast cancer dataset to study the importance of attributes. By using Weighted Association Rule Mining (WARM) more interesting rules were obtained from two attributes, multi attributes, and with provided class label using the following formulas.

#### 3.4.1. Two Attributes WARM

In this weighted support between two attributes are calculated using minimum support value = 36% and weighted confidence value = 70% as shown in Table 3 using the formula designed in paper [24] to generate strong rules and are also mentioned in formulas (3) and (4).

(3) $$\begin{array}{c} WS\left(A1\to A2\right)=\frac{\text{Summatiom of tuples of weights having given}\left(\text{attribute,value}\right)\text{pair}}{\text{Total weights of all tuples}}, \end{array}$$

(4) $$\begin{array}{c} WS\left(A1\to A2\right)=\frac{WS\left(A1⟶A2\right)}{WS\left(A1\right)}. \end{array}$$

#### 3.4.2. Multiattribute WARM

In the weighted support between multi attributes are calculated using WARM with resetting minimum support threshold value support of 36% and confidence of 70% as shown in Table 3 and then weighted confidence between multiattributes are calculated using the formula proposed in paper [24] to generated strong rules and also mentioned in formulas (5) and (6).

(5) $$\begin{array}{c} WS\left(A1\to A2,A3\right)=\frac{\sum_{i=1}^{A1}\sum_{j=1}^{A2}\sum_{k=1}^{A3}W\left(rijk\right)}{\sum_{k=1}^{n}W\left(rk\right)}, \end{array}$$

(6) $$\begin{array}{c} WS\left(A1\to A2,A3\right)=\frac{WS\left(A1⟶A2,A3\right)}{WS\left(A1\right)}. \end{array}$$

#### 3.4.3. Class_Label WARM

Formulas defined in paper [24] are used to calculate weighted support and weighted confidence by resetting the minimum support threshold value with a given class label as mentioned in formulas (7) and (8).

(7) $$\begin{array}{c} WS\left(X\to \text{Class}\_\text{label}\right)=\frac{\text{Summation of attribute set weights of the antecedent of the rule with a given class label}}{\text{Total Weights of all records}}, \end{array}$$

(8) $$\begin{array}{c} WS\left(X->\text{Classlabel}\right)=\frac{WS\left(X⟶\text{Classlabel}\right)}{WS\left(X\right)}. \end{array}$$

Using formulas (7) and (8), the knowledge base rules will be generated based on support and confidence and according to the algorithm proposed, the next step is to calculate weighted Bayes confidence (WBC) and weighted Bayes lift (WBL).

#### 3.4.4. Weighted Bayes Confidence

The formula of WBC of the rule *A* → *B* is depicted as *P*(*B|A*) which is assessed using BN as in the following equation:

(9) $$\begin{array}{c} \text{WBC}\left(A\to B\right)=\frac{\text{WS}\left(A,B\right)}{\text{WS}\left(A\right)}. \end{array}$$

#### 3.4.5. Weighted Bayes Lift

WBL for a given rule *A* → *B* is defined as WBC/*P*(*B*) as shown in (10) [24]. Using these two calculating measures strong rules are generated for building the WBBN model.

(10) $$\begin{array}{c} \text{WBL}=\frac{\text{WBC}}{P\left(B\right)}. \end{array}$$

## 4. Results and Discussion

In the process to assess the effectiveness of the WBBN model using weighted Bayesian association rules, a benchmark breast cancer medical dataset was used. Java version 1.8 was used to build the front end of the model while the backend was done with MySql 8. In training the new predictive model, different distributions of the dataset were used for training and testing and also variations in support and confidence as mentioned in Table 3. Here, the WBBN model was rigorously trained and tested on a different distribution of breast cancer dataset of 683 records as shown in Table 3.

### 4.1. Minimum Support Threshold Setup

The importance of the Minimum support (MS) threshold on the accuracy of WBBN as MS has a direct effect on classifier model results. This is because when MS is set too low some irrelevant rules can be included in the rule base and if MS is placed too high then some useful and important generated rules with high confidence may be dropped off [19]. In this current experiment with breast cancer dataset, setting up of MS with different values is done and rigorously implemented software is trained and tested. At very first association rules are generated on the basis of weighted support and weighted confidence between two attributes, multiattributes, and with a class label. Every result is mentioned in Table 3 and then based on WBC and WBL strong rules are generated to build the WBBN model.

Comparing the proposed model, WBBN with different partition sets of breast cancer datasets with varying minimum threshold values, results are produced which is tabulated in Table 3. The WBBN was trained with a 100% dataset and tested on a 100% dataset with minimum support value = 36% and confidence = 70%, the number of strong association rules generated based on WBC and WBL was 10 and when these 10 rules were used to construct model, it predicted the accuracy of 97.08%. Again on changing the training dataset to 80% and the test dataset to 20% with the same threshold value, 7 strong rules are generated to give an accuracy of 95.7%. Next, on changing the training dataset to 70% and testing the dataset to 30% with 5 strong rules, the accuracy evaluated is 97.18%. And, on rigorous computation on setting up different threshold values and varying dataset partition, the proposed model is evaluated and its empirical results were studied and tabulated in Table 3. One of the elementary performance measures of the predictive algorithm is accuracy through rigorous study of the whole dataset of 683 records. And then partitioning the dataset in different percentages and also with different minimum threshold values. The highest accuracy achieved for WBBN when trained on 478 records of the dataset and tested on 205 records of the dataset was 97.18% with support = 36% and confidence = 70%.

### 4.2. Construction of Weighted Bayesian Belief Network Classifier Using Open Markov

**Figure 2:**
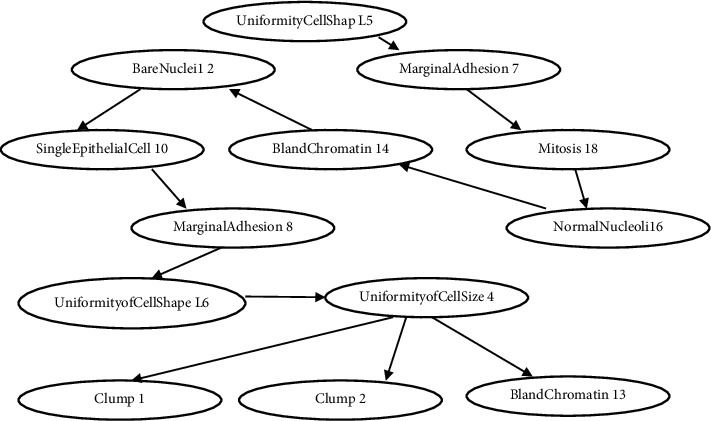
WBBN Model using Open Markov.

Open Markov is used to build the novel classifier [14]. It is used for probabilistic graphical models such as Bayesian network, influence diagram which is developed for making intelligent decision support system. Open Markov is designed to learn Bayes Net from the dataset interactively. It is a very cost-effective way of analysis [14]. In the proposed model, the K2 algorithm is used to construct the WBBN automatically provided with generated strong 5 rules with the highest WBC and WBL as mentioned in Table 4. In Open Markov, for parametric learning different values can be used with value *α* = .5 is set to learn the model's numeric probabilities. Using OpenMarkov, the network was generated known as Weighted Bayesian belief network (WBBN) as shown in Figure 2. Using this model the clinicians can draw useful information like for example Uniformity of CellSize4 attribute has a direct impact on clump1 and clump2 attributes as UniformityofCellSize4 can be calculated using these two values. Again from the network MarginalAdhesion8 (loss of adhesion) has a direct impact over Uniformity of CellShape6 which is quite intuitive. Figure 2 shows the WBBN model using Open Markov.

## 5. Comparative Studies

Again the proposed model is applied on four more different clinical datasets from the UCI repository and its discretized form is downloaded from Liverpool University (LUCS-KDD DATASET in.num format) for rigorous comparisons and the results are very outstanding as WBBN performance is at the higher end which proves that the proposed algorithm based model Weighted Bayesian Belief network works efficiently with varieties of clinical datasets as shown in Table 4. This table shows the highest accuracy attained by setting different minimum threshold values for support and confidence with different ratios of training and testing dataset.

Table 5 shows the accuracy comparison of the proposed Weighted Bayesian Belief Network with other state of the art systems like naïve Bayes [26], SVM [27], MLP [28], KNN [27], Decision Tree [29], CART [30], WCBA [7], and other algorithms [36–42, 45]. The comparative analysis indicates that both the proposed system and idea outperformed other systems in overall accuracy for the Breast cancer Wisconsin Dataset from the UCI repository. In Table 6 the research work done by various authors on the Bayesian network on the same Wisconsin breast cancer dataset is mentioned [2, 31–33] is shown and it is found that the proposed Weighted Bayesian Belief network outperformed in terms of accuracy (97.18%) on the basis of exhaustive study. Also, Table 6 shows the comparison of the proposed model with existing Bayesian models on the WBC dataset. The experimental results confirmed that the Weighted Bayesian belief network model is more authenticate and well founded than other existing models and can be used for breast cancer diagnosis and its improvement.

## 6. Conclusions and Future Work

Weighted Bayesian belief network modeled for predictive modeling not only gives very satisfactory results when applied on breast cancer clinical dataset but also with other clinical datasets like Hepatitis, Diabetics, Liver disorder, and Heart from UCI machine learning repository to show that it not only works well with one type of medical data. Hence it is a much more generalized network model with high accuracy and a low error rate. Through a comprehensive study of the model it proves that this proposed methodology is a novel investigation in the field of Bayesian network which will definitely help the medical community and other area to excel. In the future, we intend to design an intelligent App to provide support for people diagnosed with breast cancer and also more awareness about the disease, particularly in the rural areas. [34–43].

## Figures and Tables

**Table 1:** Breast Cancer Dataset with discretized values.

S.No	Attribute_name	Range
1	Clump _thickness [1–10]	1
		2

2	Uniformity_of_cellsize [1–10]	3
		4

3	Uniformity_of_cellshape [1–10]	5
		6

4	Marginal_Adhesion [1–10]	7
		8

5	Single_Epithelial_cellsize [1–10]	9
		10

6	Bare_Nuclei [1–10]	11
		12

7	Bland_Chromatin [1–10]	13
		14

8	Normal_Nucleoli [1–10]	15
		16

9	Mitoses [1–10]	17
		18

10	Output (class label representing 2 typesof breast cancer class)	19
		20

**Table 2:** Weights of attribute, value pair with class label = ‘yes'.

Attribute, value pair	Rank	Weights (class label = Yes)
Clump Thickness,1	18	.009
Clump Thickness,2	10	.11
UniformityofCellSize,3	19	.002
UniformityofCellSize,4	9	.12
UnformityofCellShape,5	20	.001
UnformityofCellShape,6	8	.095
MarginalAdhesion,7	17	.015
MarginalAdhesion,8	11	.107
SingleEpithlialCell,10	7	.122
BareNuclei,12	7	.122
BlandChromatin,13	21	.001
BlandChromatin,14	8	.121
NormalNuclei,16	7	.122
Mitosis,17	7	.122

**Table 3:** Generation of strong rules on basis of WBC and WBL and its accuracy.

S.No	Minimum threshold	Training dataset (%)	Testing dataset (%)	No. of association rules based on weighted support and weighted confidence	No. of strong association rules based on WBC and WBL	Accuracy (%)
1	Support = 36% Confidence = 70%	100	100	22	10	97.08
2		80	20	11	7	95.7
3		70	30	11	5	97.18
4		60	40	11	7	92.5

5	Support = 40% Confidence = 80%	100	100	11	7	89.53
6		80	20	11	7	95.74
7		70	30	11	7	86
8		60	40	28	12	92.55

9	Support = 26% Confidence = 60%	100	100	23	11	89.53
10		80	20	22	11	95.74
11		70	30	23	12	97.18
12		60	40	11	9	92.55

13	Support = 10% Confidence = 50%	100	100	23	12	89.53
14		80	20	23	12	95.74
15		70	30	23	12	97
16		60	40	23	12	92.5

**Table 4:** Performance of WBBN on various Clinical Datasets.

Datasets	No. ofclass labels	Minimum threshold	Trainingdataset (%)	Testingdataset (%)	No. of strong association rulesbased on WBC and WBL	Accuracy (%)
Heart	5	Support = 36%	70	30	7	92.7
		Confidence = 70%				

Pima indian	1	Support = 40%	80	20	7	95.8
		Confidence = 80%				

Hepatitis	2	Support = 36%	70	30	6	94.18
		Confidence = 70%				

Liver disorder	2	Support = 40%	70	30	5	94.3
		Confidence = 80%				

**Table 5:** Comparison of proposed model and other models applied to WBC Data set.with existing Bayesian models on WBC.

Dataset	Model	Accuracy (%)
Wisconsin Breast Cancer Dataset	Weighted Bayesian Belief Network	97.18
	Naive bayes	95.99
	SVM	97.13
	MLP	95.27
	KNN	95.27
	Decision tree	95.36
	CART	93.3
Bayesian Network		96.5

**Table 6:** Comparison of proposed model with existing Bayesian models on WBC dataset.

Model	Dataset	Accuracy (%)
WBBN	WBC	97.18
Bayesian Network		96.1
Bayes Net		95.25
Bayesian Network		96.5

## Data Availability

The cancer datasets used in this proposal are extracted from the University of California Irvine machine learning repository. UCI machine learning breast cancer dataset.” http://csc.liv.ac.uk/∼frans/KDD/software/LUCS-KDDDN/datasets/dataSet.html.
